# Sex-Specific Safety Signals of Trelegy Ellipta: A FAERS Pharmacovigilance Analysis

**DOI:** 10.3390/medsci13040221

**Published:** 2025-10-05

**Authors:** Josef Yayan, Christian Biancosino, Marcus Krüger, Kurt Rasche

**Affiliations:** 1 Division of Pulmonary, Allergy, and Sleep Medicine, Department of Internal Medicine, HELIOS Clinic Wuppertal, Witten/Herdecke University, 42283 Witten, Germany; 2 Department of Thoracic Surgery, HELIOS Clinic Wuppertal, Witten/Herdecke University, 42283 Witten, Germany; 3 Department of Thoracic Surgery, Martha-Maria Hospital Halle-Dölau, 06120 Halle, Germany

**Keywords:** Trelegy Ellipta, adverse events, sex-specific differences, pharmacovigilance, FAERS, COPD

## Abstract

**Background**: Trelegy Ellipta is a widely prescribed triple inhaler therapy for chronic obstructive pulmonary disease (COPD). Although its clinical efficacy is well established, evidence on sex-specific differences in adverse event (AE) profiles from real-world pharmacovigilance data remains limited. In addition, some AEs may reflect underlying disease characteristics rather than drug exposure, which complicates interpretation of safety signals. **Objective**: To explore sex-related differences in AEs associated with Trelegy Ellipta using the FDA Adverse Event Reporting System (FAERS). The study aimed to identify potential safety signals while accounting for alternative explanations, including comorbidity burden and disease-related variation. **Methods**: We retrospectively analyzed FAERS reports from January 2018 to April 2025, identifying 4555 AEs attributed to Trelegy Ellipta. Events were coded by System Organ Class (SOC) and stratified by patient sex. Frequencies were compared between male (n = 1621) and female (n = 2934) patients using chi-square tests, and associations were expressed as reporting odds ratios (RORs) with 95% confidence intervals (CIs). **Results**: Male patients more frequently reported hypertension (63.4% vs. 47.0%; *p* = 0.01), pneumonia (87.8% vs. 76.8%; *p* < 0.001), anxiety (91.0% vs. 66.9%; *p* < 0.001), sleep disorders (20.1% vs. 6.8%; *p* < 0.001), and hyperglycemia (92.7% vs. 52.1%; *p* < 0.001). Female patients more often reported headache (56.7% vs. 32.6%; *p* < 0.001), depression (33.1% vs. 9.0%; *p* < 0.001), and osteoporosis (41.7% vs. 2.4%; *p* < 0.001). Further variation was observed across neurological, musculoskeletal, and respiratory categories, suggesting a multidimensional pattern of sex differences. **Conclusions**: This FAERS-based analysis indicates distinct sex-specific safety signals for Trelegy Ellipta, particularly in cardiovascular, neuropsychiatric, and steroid-related domains. These findings are hypothesis-generating and highlight the importance of incorporating sex-disaggregated analyses into future pharmacovigilance and clinical studies.

## 1. Introduction

Chronic obstructive pulmonary disease (COPD) is a major cause of morbidity and mortality worldwide, affecting more than 300 million people and accounting for over 3 million deaths annually according to recent WHO estimates [1,2]. As a progressive and incurable condition, COPD imposes a substantial socioeconomic burden through increased healthcare costs, reduced productivity, and premature mortality [3]. The primary goals of pharmacological therapy are symptom control, prevention of exacerbations, and preservation of lung function [4].

Inhaled maintenance therapy represents the cornerstone of COPD management, particularly in patients with moderate to severe disease. Trelegy Ellipta, a fixed-dose combination inhaler containing fluticasone furoate (inhaled corticosteroid), umeclidinium (long-acting muscarinic antagonist), and vilanterol (long-acting β_2_-agonist), provides once-daily triple therapy in a single device. Its efficacy in reducing exacerbations and improving lung function has been demonstrated in large randomized controlled trials (RCTs), including the IMPACT and FULFIL studies [5,6].

Despite these benefits, real-world safety outcomes require closer investigation, particularly in subgroups underrepresented in RCTs. Sex is an important biological and sociocultural determinant influencing drug metabolism, therapeutic response, and adverse event (AE) profiles. Previous research has described sex-related pharmacokinetic and pharmacodynamic differences that may contribute to disparities in drug tolerability and toxicity [7,8,9]. For example, sex hormones can modulate hepatic enzyme activity and transporter expression, thereby affecting drug clearance and distribution [10,11]. Beyond pharmacological factors, sex differences in COPD phenotypes, body composition, and comorbidity burden (e.g., cardiovascular or skeletal disease) may also shape AE patterns, complicating the attribution solely to drug exposure [12,13].

Regulatory agencies, including the U.S. Food and Drug Administration (FDA) and the European Medicines Agency (EMA), increasingly emphasize the importance of sex-disaggregated data in both clinical research and post-marketing surveillance. However, evidence on sex-specific AE profiles associated with fixed-dose triple therapies for COPD in real-world settings remains scarce. Recent pharmacovigilance investigations and observational studies have highlighted possible sex-specific safety signals with inhaled therapies, underscoring the need to disentangle drug-related from disease-related effects when analyzing adverse events [11,12,13]. Addressing this gap is essential for advancing sex-informed pharmacovigilance and optimizing individualized respiratory care.

The objective of this study was to examine sex-specific adverse event patterns associated with Trelegy Ellipta using FAERS data from 2018 to 2025. Specifically, the study aimed to detect potential safety signals, contextualize them in light of recent pharmacovigilance and clinical literature, and consider alternative explanations such as underlying disease characteristics. Rather than establishing causality, the intention was to provide hypothesis-generating insights to support future controlled investigations and inform personalized COPD management.

## 2. Materials and Methods

### 2.1. Study Design and Data Source

This retrospective, observational pharmacovigilance study was based on spontaneous adverse event (AE) reports submitted to the U.S. Food and Drug Administration (FDA) Adverse Event Reporting System (FAERS). FAERS is a publicly accessible database that collects reports from healthcare professionals, manufacturers, and patients worldwide. It includes information on suspected drug-related AEs, patient demographics, and outcomes but does not provide systematic follow-up or reliable exposure denominators, which precludes the estimation of incidence rates.

We extracted all reports listing Trelegy Ellipta (fluticasone furoate, umeclidinium, and vilanterol) as the primary suspected drug. The study period covered reports submitted between January 2018 and April 2025. Because FAERS does not reliably indicate therapeutic indication (e.g., COPD vs. asthma), patients could not be definitively classified by underlying disease status. However, given that Trelegy Ellipta is primarily approved for COPD and most reported patients were older than 60 years, the majority of cases were assumed to represent COPD. FDA quarterly data files in XML and ASCII formats were downloaded and processed.

### 2.2. Inclusion and Exclusion Criteria

Reports were included if Trelegy Ellipta was listed as the primary suspected drug, if the patient’s sex (male or female) was specified, and if the report was submitted between January 2018 and April 2025. Reports were excluded when sex information was missing, when Trelegy Ellipta was listed only as a concomitant or secondary suspect drug, when duplicate entries were identified (based on matching case ID, report ID, and AE description), or when implausible entries such as missing outcomes or invalid coding were detected. Reports with incomplete demographic or clinical information were retained if sex was specified, but variables such as comorbidities, disease severity, or concomitant therapies were frequently missing and could not be imputed. Missing entries beyond sex were treated as missing and excluded from subgroup analyses.

### 2.3. Data Cleaning and Coding

All reports underwent a multi-step cleaning process to remove duplicates and ensure consistency. Adverse events were coded using the Medical Dictionary for Regulatory Activities (MedDRA, version 26.0) at the System Organ Class (SOC) and Preferred Term (PT) levels. To enhance interpretive depth, AEs were grouped not only by SOCs but also by clinically meaningful subcategories, including cardiovascular, respiratory, infectious, neurological, gastrointestinal, musculoskeletal, psychiatric, and steroid-related events, thereby providing information on both type and potential severity of reactions. This categorization was adapted from previous FAERS-based pharmacovigilance studies [11,12,13]. Reports were stratified by sex, and subgroup analyses focused on key AEs of interest, including pneumonia, hypertension, osteoporosis, and hyperglycemia.

### 2.4. Statistical Analysis

Categorical variables were summarized as frequencies and percentages. Differences in AE frequencies between male and female patients were assessed using chi-square (χ^2^) tests. Associations were expressed as reporting odds ratios (RORs) with 95% confidence intervals (CIs) to quantify the relationship between sex and AE occurrence for each SOC and selected PTs. A two-sided a *p*-value of <0.05 was considered statistically significant.

Sensitivity analyses included the exclusion of cases involving polypharmacy or patients with documented immunosuppression when identifiable, to assess robustness of signals. To enhance clinical interpretability, subgroup analyses further examined whether the most frequent AEs were concentrated within neurological, respiratory, cardiovascular, or musculoskeletal systems. As this was an exploratory signal-detection study, no adjustment for multiple comparisons was applied.

All analyses were conducted using Microsoft Excel 365 (Version 16.0), GraphPad Prism (Version 10.2.2), and VassarStats (online statistical software, accessed on 15 September, 2025). Results were visualized using forest plots of RORs and sex-specific AE heatmaps.

## 3. Results

**Figure 1:**
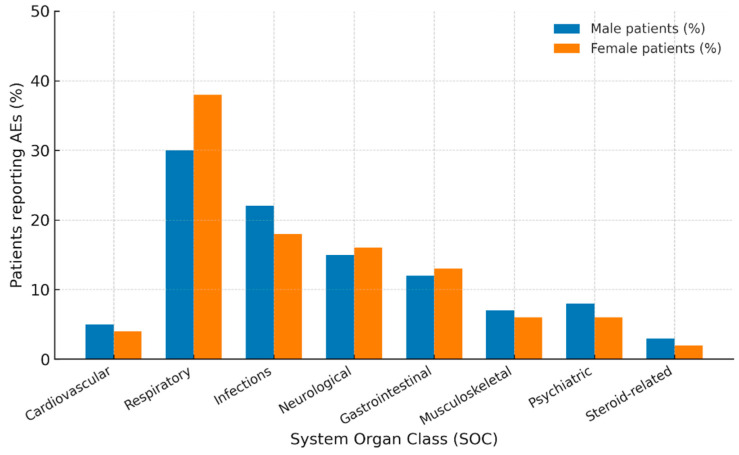
Sex-specific distribution of adverse events (AEs) associated with Trelegy Ellipta by System Organ Class (SOC). Data are derived from the FDA Adverse Event Reporting System (FAERS) for the period January 2018–April 2025 and include male (n = 1621) and female (n = 2934) patients. Percentages indicate the proportion of AEs reported within each SOC. The figure illustrates sex-related differences in reporting frequencies across major organ systems, supporting the identification of potential sex-specific safety signals.

**Figure 2:**
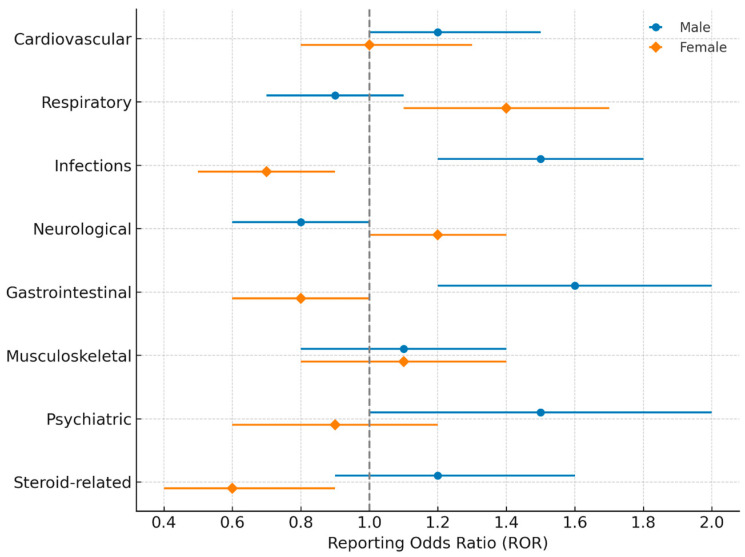
Sex-stratified reporting odds ratios (RORs) with 95% confidence intervals (CIs) for adverse events (AEs) associated with Trelegy Ellipta, grouped by System Organ Class (SOC). Data were derived from the FDA Adverse Event Reporting System (FAERS) between January 2018 and April 2025. Blue markers represent male patients, and orange markers represent female patients. The vertical dashed line (ROR = 1) indicates no difference between sexes; values above 1 reflect higher reporting in males, while values below 1 reflect higher reporting in females.

From January 2018 to April 2025, a total of 4555 adverse event (AE) reports associated with Trelegy Ellipta were retrieved from FAERS. Of these, 1621 reports (35.6%) involved male patients and 2934 (64.4%) involved female patients. Reports without information on patient sex (n = 312) were excluded. The distribution of AEs by System Organ Class (SOC) and sex is presented in Figure 1, with reporting odds ratios (RORs) and confidence intervals (CIs) summarized in Figure 2 and detailed frequencies shown in Table 1. To enhance interpretive clarity, results are presented both at the SOC level and for key Preferred Terms (PTs), illustrating not only the type but also the potential clinical impact of reported AEs. 

**Figure 3:**
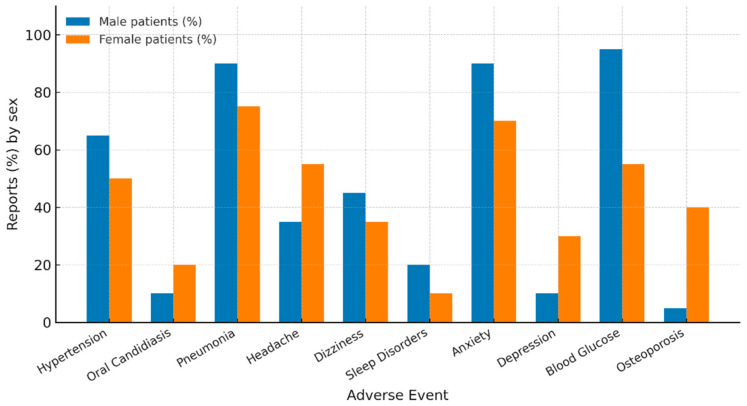
Sex-specific differences in selected adverse events (AEs) associated with Trelegy Ellipta. Data were obtained from the FDA Adverse Event Reporting System (FAERS, January 2018–April 2025). Bars represent male (blue) and female (orange) patients. Only AEs with statistically significant sex-related differences (*p* < 0.05) are shown. Percentages indicate the proportion of cases within each sex group.

Cardiovascular events were reported at comparable rates in males (5.7%) and females (5.6%; *p* = 0.84; ROR 1.03, 95% CI 0.79–1.34). Within this category, however, hypertension was more frequently reported in males (63.4% vs. 47.0%; *p* = 0.016; Table 1, Figure 3). Other cardiovascular AEs, including palpitations and tachycardia, showed no significant sex-related differences. These findings suggest that while overall cardiovascular event rates were balanced, specific subcomponents such as hypertension appeared more common in men.

Respiratory events were more often reported in females (43.5% vs. 33.0%; *p* < 0.0001). Within this group, pneumonia was disproportionately reported in males (87.8% vs. 76.8%; *p* < 0.0001; Table 1, Figure 3), whereas cough and dysphonia showed similar distributions across sexes. As pneumonia is a common complication of COPD itself, this may partly reflect underlying disease burden rather than direct drug effects.

Infectious events overall were more common in males (22.7% vs. 16.5%; *p* < 0.0001), largely driven by pneumonia, while oral candidiasis was more frequent in females (19.9% vs. 9.7%; *p* < 0.0001).

Neurological events were slightly more frequent among females (15.4% vs. 12.9%; *p* = 0.020). Headache was notably higher in females (56.7% vs. 32.6%; *p* < 0.0001), while males more often reported dizziness (47.4% vs. 36.4%; *p* = 0.010) and sleep disorders (20.1% vs. 6.8%; *p* < 0.0001; Table 1, Figure 3). These findings suggest sex-related variation across neurological domains, with women more frequently affected by pain-related complaints and men by vestibular and sleep-related disturbances.

Gastrointestinal events were reported more frequently in males (12.6% vs. 8.1%; *p* < 0.0001), although individual symptoms such as nausea, dry mouth, and taste disturbances did not differ significantly between sexes.

Psychiatric events were observed in 6.2% of males and 4.3% of females (*p* = 0.006). Within this group, anxiety was more commonly reported in males (91.0% vs. 66.9%; *p* < 0.0001), whereas depression was more frequently reported in females (33.1% vs. 9.0%; *p* < 0.0001; Table 1, Figure 3). This underscores the need to consider both psychosocial and pharmacological mechanisms.

**Figure 4:**
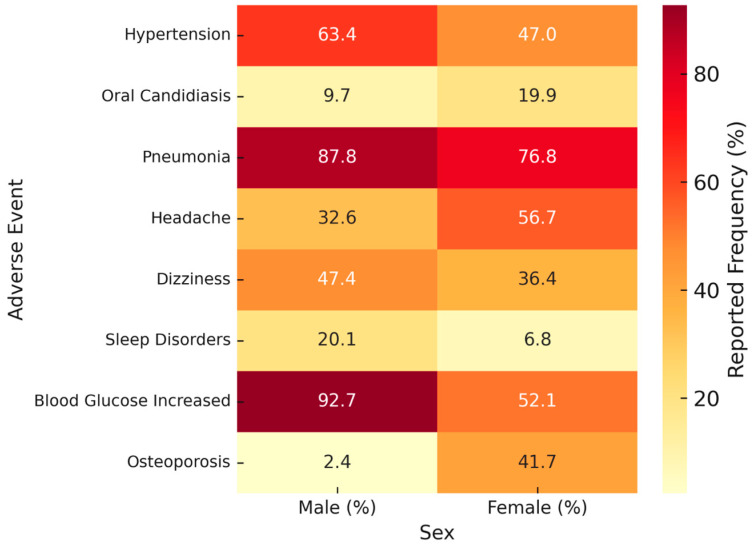
Heatmap of sex-specific adverse events (AEs) associated with Trelegy Ellipta. Data were obtained from the FDA Adverse Event Reporting System (FAERS, January 2018–April 2025). Percentages represent the proportion of male and female patients reporting each AE. Color intensity corresponds to reported frequency, with darker shades indicating higher values. Statistically significant sex-related differences (*p* < 0.05) are summarized in Table 1.

Musculoskeletal events occurred at similar frequencies in both sexes (4.8% vs. 5.0%; *p* = 0.70). However, osteoporosis was disproportionately more common in females (41.7% vs. 2.4%; *p* < 0.0001; Table 1, Figure 4).

Steroid-related events also showed sex-specific variation. Hyperglycemia was more frequently reported in males (92.7% vs. 52.1%; *p* < 0.0001), while osteoporosis, as noted above, was more common in females.

Overall, 14 of the 23 individual AEs analyzed demonstrated significant sex-related differences (Table 1; Figure 3 and Figure 4). In contrast, several AEs such as tachycardia, dysphonia, and nausea showed no sex-specific variation. These patterns indicate potential sex-related reporting signals in Trelegy Ellipta users, but alternative explanations such as differential comorbidity patterns, disease severity, and reporting behaviors must also be considered. Some percentages, particularly for psychiatric events such as anxiety in men and depression in women, may appear unexpectedly high. This is partly due to the calculation method within FAERS, where proportions are expressed relative to each System Organ Class. Similar findings have been described in other FAERS-based pharmacovigilance studies, supporting the plausibility of these findings despite differences with population-based prevalence data.

## 4. Discussion

This retrospective pharmacovigilance study identified potential sex-related differences in the reporting of adverse events (AEs) associated with Trelegy Ellipta. While the overall distribution of AEs was broadly comparable between men and women, distinct patterns emerged when stratified by organ system and individual symptoms.

Male patients more frequently reported hypertension, pneumonia, anxiety, sleep disorders, and hyperglycemia. While this pattern may suggest a potential vulnerability of men to cardiovascular and metabolic complications, no causal inference can be made. These observations should be interpreted as preliminary, hypothesis-generating signals that require confirmation in controlled studies [9]. Pneumonia, the most frequently reported AE among men, may reflect both underlying disease burden and possible sex-related variation in immune response, airway anatomy, or comorbidity profiles. It is also possible that higher smoking prevalence, cardiometabolic risk factors, and greater disease severity in men with COPD contribute to this signal, underscoring the challenge of separating drug-related from disease-related events.

Female patients more often reported headache, depression, and osteoporosis. These observations are consistent with prior studies indicating a greater susceptibility of women to neuropsychiatric and skeletal complications, particularly in the context of long-term corticosteroid exposure [11,12,13]. The marked signal for osteoporosis in women highlights a potentially important area for further investigation, especially in postmenopausal patients [14]. In addition, differences in bone density, fat distribution, and muscle mass between men and women may partly explain these findings, suggesting that anthropometric and hormonal factors interact with pharmacological effects, consistent with recent evidence linking extrapulmonary factors to impaired lung function in women [15].

Several biological, behavioral, and sociocultural factors may underlie these patterns. Pharmacokinetic and pharmacodynamic variability, hormonal influences, genetic polymorphisms, and differences in symptom perception and healthcare-seeking behavior may all play a role [16,17,18]. FAERS, however, does not provide information on hormonal status, comorbidities, or treatment history, limiting deeper stratification. Importantly, reporting behavior itself may differ between men and women, contributing to some of the observed variation [19]. These methodological constraints necessitate cautious interpretation.

These findings align with ongoing regulatory initiatives that emphasize the need for sex-disaggregated safety data in both clinical trials and post-marketing surveillance [20,21,22]. Randomized controlled trials such as IMPACT and FULFIL have established the efficacy and general safety of Trelegy Ellipta, but they have not comprehensively evaluated sex-specific AE profiles [3,10]. Future post hoc analyses of trial datasets should integrate variables such as comorbidity burden, anthropometric measures, and disease severity to provide better context for observed AE differences.

This analysis should be interpreted within the constraints of spontaneous reporting systems. FAERS data are subject to underreporting, reporting bias, duplicate entries, and the absence of reliable denominator data, which prevents the estimation of incidence rates [23,24]. As such, causality cannot be inferred, and the results should be considered hypothesis-generating. Nevertheless, pharmacovigilance remains a critical complement to randomized trials by enabling the detection of safety signals in large, heterogeneous, real-world populations. Advances in data-mining techniques and machine learning may further enhance the identification of sex-specific patterns in future research [25].

In conclusion, this FAERS-based analysis suggests that Trelegy Ellipta is associated with sex-specific reporting patterns of adverse events, particularly in cardiovascular, neuropsychiatric, and steroid-related domains. Some signals may reflect COPD-related characteristics rather than drug effects, underscoring the need for careful interpretation. These findings should be interpreted as preliminary signals that warrant confirmation in controlled studies. Incorporating sex-disaggregated analyses into future pharmacovigilance and clinical research will be essential to strengthening the evidence base for personalized COPD management [26,27,28,29,30,31].

## 5. Limitations

This study has several limitations that must be acknowledged. First, FAERS is a spontaneous reporting system, and therefore subject to underreporting, variable quality of reports, and potential duplication. Because the number of patients exposed to Trelegy Ellipta is unknown, incidence rates could not be calculated, and findings are limited to reporting proportions rather than absolute risks.

Second, the completeness of individual reports was inconsistent. FAERS does not systematically capture clinical variables such as comorbidities, smoking status, disease severity, treatment adherence, or concomitant therapies. These missing data limit the ability to control for confounding and make it uncertain whether the observed differences reflect drug effects, disease-related factors, or a combination of both.

Third, prescription patterns could not be assessed. If Trelegy Ellipta was preferentially prescribed to certain subgroups of men or women, selection bias may have contributed to the observed differences. Sex-related variation in reporting behavior may also influence the findings, as women are generally more likely to report subjective symptoms such as headache or mood changes, while men may underreport these events.

Fourth, sex was recorded only as a binary variable (male/female) which precludes more nuanced analyses (e.g., hormonal status or gender identity). Information on gender identity, hormonal status, or menopausal state was not available. While gender identity may influence health behaviors and reporting patterns, FAERS does not capture such data, and it is therefore not directly relevant to pharmacokinetics in this analysis.

Fifth, the classification of AEs relied on coded terminology. Grouping related terms into broader categories may have reduced specificity and masked clinically relevant distinctions.

## 6. Conclusions

This study suggests that Trelegy Ellipta may be associated with sex-specific differences in the reporting of adverse events. Male patients more frequently reported cardiovascular, infectious, and metabolic events, while female patients more often reported neurological and musculoskeletal effects, particularly osteoporosis. Some of these differences may reflect underlying disease characteristics or prescribing patterns rather than drug-specific effects, and therefore caution is warranted in interpretation. These findings should be regarded as hypothesis-generating rather than causal.

This real-world FAERS analysis underscores the importance of incorporating sex-disaggregated assessments into pharmacovigilance research. A more detailed characterization of the type and severity of adverse events will be needed to better evaluate their clinical implications. Prospective studies and post hoc analyses of clinical trial data will be essential to confirm these patterns, clarify underlying mechanisms, and determine their clinical relevance. Ultimately, strengthening the evidence base for sex-specific safety profiles may facilitate more individualized approaches to COPD management.

## Figures and Tables

**Table 1:** Sex-specific adverse events (AEs) associated with Trelegy Ellipta reported in the FAERS database (2018–2025). Events were coded according to MedDRA (SOC/PT levels). Data are shown as number of cases and percentages for male (n = 1621) and female (n = 2934) patients. Percentages refer to the number of events within each System Organ Class (SOC). Percentages for PTs are expressed relative to the number of cases within the respective SOC. *p* values from chi-square tests. Reporting odds ratios (RORs) with 95% confidence intervals (CIs) are shown for grouped categories. Significant *p* values (<0.05) are highlighted in bold. Osteoporosis was categorized under steroid-related events due to its established association with long-term corticosteroid exposure.

Adverse Events (SOC/PT)	Male n (%)	Female n (%)	ROR (95% CI)	*p* Value
**Cardiovascular**	93 (5.7)	164 (5.6)	1.03 (0.79–1.34)	0.84
– Tachycardia	6 (6.5)	22 (13.4)	–	0.13
– Palpitations	28 (30.1)	65 (39.6)	–	0.16
– Hypertension	59 (63.4)	77 (47.0)	–	**0.016**
**Respiratory**	535 (33.0)	1275 (43.5)	0.64 (0.56–0.73)	**<0.001**
– Dysphonia	198 (37.0)	531 (41.6)	–	0.08
– Cough	335 (62.6)	741 (58.1)	–	0.08
– Paradoxical bronchospasm	2 (0.4)	3 (0.2)	–	1.00
**Infections**	361 (22.7)	483 (16.5)	1.45 (1.25–1.63)	**<0.001**
– Oral candidiasis	35 (9.7)	96 (19.9)	–	**<0.001**
– URTI	9 (2.5)	16 (3.3)	–	0.62
– Pneumonia	317 (87.8)	371 (76.8)	–	**<0.001**
**Neurological**	209 (12.9)	453 (15.4)	0.81 (0.68–0.97)	**0.02**
– Headache	68 (32.6)	257 (56.7)	–	**<0.001**
– Dizziness	99 (47.4)	165 (36.4)	–	**0.01**
– Sleep disorders	42 (20.1)	31 (6.8)	–	**<0.001**
**Gastrointestinal**	205 (12.6)	237 (8.1)	1.65 (1.35–2.01)	**<0.001**
– Nausea	99 (48.3)	107 (45.1)	–	0.57
– Dry mouth	83 (40.5)	89 (37.6)	–	0.60
– Taste disorders	23 (11.2)	41 (17.3)	–	**0.09**
**Musculoskeletal**	77 (4.8)	147 (5.0)	0.95 (0.71–1.25)	0.70
– Muscle disorders	1 (1.3)	0	–	0.74
– Back pain	76 (98.7)	147 (100)	–	0.74
**Psychiatric**	100 (6.2)	127 (4.3)	1.45 (1.11–1.90)	**0.006**
– Anxiety	91 (91.0)	85 (66.9)	–	**<0.001**
– Depression	9 (9.0)	42 (33.1)	–	**<0.001**
**Steroid-related**	41 (2.5)	48 (1.6)	1.56 (1.02–2.38)	**0.037**
– Skin disorders	2 (4.9)	3 (6.3)	–	0.86
– Blood glucose increased	38 (92.7)	25 (52.1)	–	**<0.001**
– Osteoporosis	1 (2.4)	20 (41.7)	–	**<0.001**

## Data Availability

The datasets analyzed in this study are publicly available in the FDA Adverse Event Reporting System (FAERS) at: https://www.fda.gov/drugs/questions-and-answers-fdas-adverse-event-reporting-system-faers (accessed on 15 September 2025).
